# Thermal, High Pressure, and Electric Field Processing Effects on Plant Cell Membrane Integrity and Relevance to Fruit and Vegetable Quality

**DOI:** 10.1111/j.1750-3841.2010.01763.x

**Published:** 2010-09

**Authors:** Maria E Gonzalez, Diane M Barrett

**Affiliations:** Dept. de Agroindustrias, Facultad de Ingeniería Agrícola, Univ. de ConcepciónVicente Méndez 595, Chillán, Region Bio Bio, Chile; Dept. Food Science and Technology, Univ. of CaliforniaDavis, CA 95616, U.S.A.

**Keywords:** high pressure, membrane integrity, plant cells, pulsed electric fields, texture

## Abstract

Advanced food processing methods that accomplish inactivation of microorganisms but minimize adverse thermal exposure are of great interest to the food industry. High pressure (HP) and pulsed electric field (PEF) processing are commercially applied to produce high quality fruit and vegetable products in the United States, Europe, and Japan. Both microbial and plant cell membranes are significantly altered following exposure to heat, HP, or PEF. Our research group sought to quantify the degree of damage to plant cell membranes that occurs as a result of exposure to heat, HP, or PEF, using the same analytical methods. In order to evaluate whether new advanced processing methods are superior to traditional thermal processing methods, it is necessary to compare them. In this review, we describe the existing state of knowledge related to effects of heat, HP, and PEF on both microbial and plant cells. The importance and relevance of compartmentalization in plant cells as it relates to fruit and vegetable quality is described and various methods for quantification of plant cell membrane integrity are discussed. These include electrolyte leakage, cell viability, and proton nuclear magnetic resonance (^1^H-NMR).

## Introduction

Fruits and vegetables are important components of the human diet and consumers today are demanding more minimally processed products that retain the organoleptic characteristics of fresh produce (Garcia and Barrett 2002). Consumers perceive fresh produce as healthier, fresher, higher quality and safer than pre-packaged produce, and higher quality but less safe than frozen or canned fruits and vegetables (UFPA 2008). There is an increased awareness of quality attributes including color, texture, flavor, and nutrient content (De Belie and others 2000; Waldron and others 2003) and products that provide convenience are free from additives and preservatives yet retain the attributes of the fresh-like product are in high demand (Rastogi and others 2007).

Modification of existing food processing techniques and/or the adoption of novel technologies that allow for production of higher quality products that are microbiologically secure (Barbosa-Cánovas and others 2005) are strategies undertaken to meet these consumer demands. Clear statements about benefits associated with a particular food or novel food processing technique (for example, impact of the technology on taste, convenience, nutritional value, magnitude of the risk the technology reduces, and effect of the technology on the environment) reduces concerns toward the food or technology and improve both its acceptance and the likelihood of consumption (Bruhn 2007).

Knowledge of cell structure changes that occur as a result of high pressure (HP), electric field, and thermal processing will allow for improvement of shelf life and quality of minimally processed vegetables in order to maintain “fresh-like characteristics.” Quantification of the degree of cellular disruption will allow for the comparison and optimization of preservation processes. Reports on attempts to implement physiological and biochemical principles in the industrial processing of fruit and vegetables are not common in the literature, but recent investigations have laid the foundation for this new area of research and technological innovation (Gomez Galindo and others 2007).

## Plant Cell Integrity and Relevance to Food Quality

**Figure 1:**
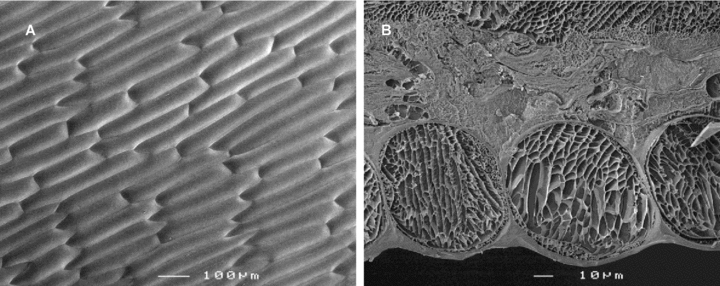
Cryogenic scanning electron micrograph of onion epidermal cells from the surface (A) and cross section (B) views.

**Figure 2:**
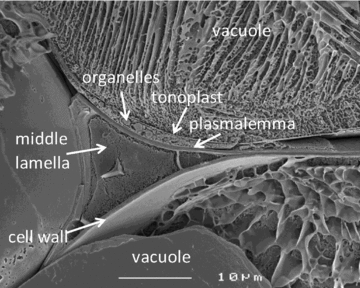
Cryogenic scanning electron micrograph of the interface between 3 different onion parenchyma cells, with various components labeled.

Fruits and vegetables represent types of plant tissues that, although they vary greatly in their biological function, are all composed of millions of cells with specialized functions and have a basic eukaryotic organization. They contain a nucleus, cytoplasm, and subcellular organelles and are enclosed in a membrane that defines their boundaries, the plasmalemma, and a cellulosic cell wall. Figure 1 and 2 are cryogenic scanning electron micrographs illustrating onion epidermal cells from surface (Figure 1A) and cross-sectional (Figure 1B) views. Figure 2 illustrates the physical separation of adjacent cells and individual “packaging” within the cell wall and plasmalemma. Mature living plant cells contain a large water filled vacuole that can occupy 80 to 90% of the total volume of the cell and is surrounded by another membrane, the tonoplast (Taiz and Zeiger 2006).

It is the presence of membrane-bound compartments or organelles within plant cells that allow biochemical reactions that are essential to life to be segregated (Figure 2). Biological membranes are bilayers composed of phospholipids that contain proteins inserted within the lipid matrix (Taiz and Zeiger 2006). Many of these proteins form channels through which the cells regulate themselves with respect to the external medium. Studies show that the extracellular membranes (plasmalemma) differ from intracellular ones (tonoplast and organelle membranes) in function, structure, and composition (Andersson and others 2003). Membranes are fluid at physiological temperatures and can lose fluidity under different conditions with a decrease in phospholipids motion. At a temperature specific to each type of lipid, the lipids undergo phase transition from liquid crystalline to a gel phase. In this gel phase, acyl chains are fully extended and phospholipid head groups are tightly packed and dehydrated (Crowe and others 1998). When gel phases and liquid phases coexist, the lipids do not pack well and increased leakage occurs. A consequence of membrane deterioration is the loss of semipermeability of the membrane, which as a diffusion barrier in the intact plant tissue (Murray and others 1989; Stanley 1991).

### Texture

Texture is one of the main attributes that govern the acceptability of a food by a consumer (Mohsenin 1986). In plant tissues, the state of the cell membranes can change from being partially to totally permeable, and this can lead to significant changes in tissue architecture (Rastogi and others 2000).

The presence of an intact plasmalemma, a semipermeable membrane, allows for the maintenance of an osmotic difference between the inside and outside of the cell. At equilibrium water potential, the osmotic difference is balanced by a positive hydrostatic pressure within the cells that is acting against the cell walls and is referred to as turgor pressure (Taiz and Zeiger 2006). Cellular turgor is an important component of the rigidity and firmness of plant materials (Ilker and Szczesniak 1990). Although the cell has different mechanisms to regulate its turgor pressure, it has been observed that it declines naturally during ripening (Shackel and others 1991) and is affected during processing (Greve and others 1994). Texture measurements can be used as an indicator of the integrity of the cell and the tissue (Rojas and others 2001; Llano and others 2003; Gonzalez and others 2010b).

### Color

Color is imparted to plant tissues by a number of water-soluble and lipid-soluble pigments. Water-soluble pigments such as the phenolics and anthocyanins are typically located either in the acidic plant cell vacuole or in the cytoplasm. Lipid-soluble pigments, on the other hand, in the intact plant tissue are found in subcellular organelles such as the chloroplasts or chromoplasts, or associated with lipid bodies or bilipid membranes. Loss of compartmentalization, due to normal senescence or processing-induced changes, may result in interaction of enzymes and substrates that affect color.

Polyphenol oxidase, for example, is the primary enzyme involved in enzymatic browning (Vámos-Vigyázó and Haard 1981) and it is initially found in the plastids, while its phenolic substrates are found in the vacuole. Barrett and others (1991) found that polyphenol oxidase activity was found in the chloroplast in freshly harvested Red Delicious apples, but during controlled atmosphere storage, the enzyme was solubilized and found to predominate in the soluble fraction of the plant cell. Fresh-cut products in particular suffer from loss of compartmentalization during cutting operations, which allow polyphenol oxidase and phenolics to interact and result in browning of cut surfaces (Garcia and Barrett 2002).

Chlorophyll bleaching, or loss or green color, is another enzymatic reaction influenced by loss of compartmentalization. Lipid-soluble chlorophyll and carotenoids such as lycopene and β-carotene may be oxidized, resulting in a loss of color, as a result of loss of compartmentalization.

### Flavor

There are a number of plant tissues that have a distinct flavor or aroma but are perceived only after loss of compartmentalization. Intact onions contain the odurless cysteine sulphoxides in their cytoplasm, but when cells are disrupted, cysteine sulphoxides are rapidly converted into (alk)enLylsulphenic acid, pyruvate, and ammonia by the enzyme alliinase (Randle and Lancaster 2002), which is initially located in the vacuole (Lancaster and Collin 1981). The corresponding thiosulphanates or the lachrymatory factor (Z)-propanethial-oxide formed by the enzymatic reaction give the characteristic smell of fresh onion juice. Off-flavor production (Paull and Chen 2000) and an environment for microbial growth (Barry-Ryan and O’Beirne 2000) have also been associated with loss of compartmentalization.

### Nutrient content

Nutrients typically found in plant tissues include vitamins and minerals as well as phytonutrients such as phenolics, glucosinolates, and carotenoids. As stated above, the carotenoids are susceptible to oxidation by lipoxygenase, which may result in a loss of nutrient content as well as color (Siedow 1991). Oxidation of phenolic compounds may not only result in a loss of color, but may also cause polymerization, and the resultant compounds may not be as biologically active (Rice-Evans and others 1996). Exposure to oxygen in general is undesirable from a nutritional point of view, and loss of tissue compartmentalization may increase availability and diffusion of oxygen into the plant tissue.

## Thermal Processing

Traditional food processing methods have relied on high temperatures as a way to ensure prolonged shelf life and food safety. However, thermal processes suffer from the limitations of heat transfer, with a gradient of temperature exposure from the outside to the inside of the food, with overprocessing causing severe damage to the sensory, nutritional, and functional properties (Butz and others 2002; San Martin and others 2002).

A number of investigators have used instead mild heat treatments to improve the shelf life quality of minimally processed products. This approach is intended to reduce the microbial load and decrease enzyme activity (Stanley and others 1995). Minimal food processing allows consumers to have fresh-like quality fruits and vegetables that are convenient to consume, but unit operations such as cutting, slicing, chopping, peeling, so on already cause loss of cellular integrity as has been discussed above, with changes in enzymatic activity, ethylene production, respiration, and accumulation of secondary metabolites (Gomez Galindo and others 2007).

### Thermal effects on microorganisms

Two levels of physical stress may be distinguished with regard to the reversibility of membrane changes in microorganisms, for example, strong and mild energy stresses (Simonin and others 2007). Exposure to high temperatures (strong stresses) can cause continuous increases in membrane permeability caused by time-dependant changes such as lipid phase transitions and protein conformation changes (Bischof and others 1995), eventually causing cell death. Membrane fluidity changes may differ significantly, according to the type of thermal stress. Simonin and others (2007) observed that a heat shock at 75 °C for 1 min in *Saccharomyces cerevisiae* induced irreversible changes in membrane fluidity, as observed by DPH (1,6 diphenyl-1,3,5-hexatriene) anisotropy. A treatment at 50 °C for 60 min in yeast cells, however, while causing cell death, also resulted in recovery of the initial membrane fluidity once the yeast cells were returned to initial conditions. In the same study, a HP treatment (300 MPa at 25 °C for 10 min) caused transient membrane perturbations similar to those observed with mild heat treatment. Cell death may then be associated with permanent modifications to the membranes as is the case with strong physical stress, but with transient and reversible modifications in the case of mild perturbations.

Guyot and others (2005) studied the mechanisms involved in slow heat gradient induced thermotolerance of *S. cerevisiae* and compared yeasts heated slowly from 25 to 50 °C at 0.5 °C/min to a rapid heat shock at 50 °C. Both conditions were maintained at this temperature for 1 h. A 50-fold higher survival rate in the slowly heated yeasts was attributed to changes in the plasma membrane properties that took place to accommodate the thermal stress. These changes did not involve protein or intracellular molecular synthesis. In contrast, in the case of the heat-shocked yeasts, a complete phospholipid disorganization led to increased membrane permeability and cell death following the heat shock.

In postharvest fruit and vegetable applications, hot water dips have been proposed as alternative approaches to chemical treatments for fungal pathogen control. Mild thermal treatments (45 °C, for 10 or 15 min or 48 °C for 5, 10 or 15 min) for decay control caused by *Botrytis cinerea* and *Monilia fructígena* proved to be effective on cherries, but not in strawberries where tissue firmness was greatly affected at these temperatures (Marquenie and others 2002). In blueberries (Fan and others 2008) investigators showed that 60 °C treatments for 30 s resulted in control of both *B. cinerea* and *Colletotrichum sp*.

### Thermal effects on plant tissues

Heating produces alterations in plant tissue microstructure that influence texture, with tissue softening brought on by loss of turgor pressure and purging of occluded air, thermal degradation of middle lamella pectins, and other cell wall polysaccharides and gelatinization of starch (Llano and others 2003).

Mild heat treatments, such as used in pasteurization or blanching, are designed to destroy pathogenic organisms in some products and to extend shelf life. Blanching in hot water (70 to 100 °C) or steam is a preliminary step to inactivate enzymes involved in quality deterioration of the processed product. Examples are steam treatments of carrot sticks that inactivated phenylalanine ammonia lyase (PAL), peroxidase (POD), and syringaldazine oxidase (SOX) and retarded surface discoloration, and formation of soluble phenolics, isocoumarins, and lignin (Howard and others 1994). Peng and Jiang (2004) found immersion of fresh-cut slices of Chinese water chestnut in boiling water for 30 s resulted in complete browning inhibition after 9 d at 4 °C associated with PAL, polyphenoloxidase (PPO), and POD activity. Mild heat treatments at 50 to 55 °C for less than 2 min resulted in improved texture and shelf life of broccoli and green peppers, and respiration was maintained following these mild heat treatments, thereby avoiding any deleterious consequences of anaerobic respiration (Yuksel and Barrett unpublished). Mild temperature treatments (70 °C, 2 min) also enhance the activity of pectin methylesterase (Anthon and Barrett 2006), resulting in increased tissue firmness as a result of a firming effect due to the cell wall component of texture.

Nevertheless, blanching treatments can have an effect on cell membranes. In kiwi fruit, blanching times of 5 min resulted in breakdown of cell membranes as observed by the fluorescein diacetate (FDA) viability test, and were closely associated with green color disappearance, a significant decrease in POD activity, and significant loss of initial and residual relaxation forces and firmness (evaluated as *F*_f_/*L*_f_; with *F*_f_= failure force, *L*_f_= failure deformation) (Llano and others 2003). Exposure time and treatment temperature play a role in membrane susceptibility (Schlüter and others 2008). A 2 min exposure to 45 °C caused a 75% reduction of the metabolic activity of fresh lettuce, measured as the maximum photochemical activity, but this pronounced reduction was reversible over a 24 h period. In contrast, an increase to 50 °C treatment caused irreversible damage to the photosynthetic apparatus, indicating transient effects of sublethal temperatures, and a narrow gap in between which reversible changes at the chloroplast membrane level occur and result in the complete loss of integrity.

Lurie and others (1997) evaluated a number of heat treatments to reduce chilling injury in tomatoes at the breaker stage. They found that hot water dips (30 min at 40 °C or 2 min at 46, 48, or 50 °C) before holding at 2 °C led to an increase in phospholipid content, a lower sterol to phospholipid ratio, and more unsaturated fatty acids relative to the unheated fruits. This can make membranes more fluid, with better selective permeability and greater responsiveness to environmental stress (Bohn and others 2001; Zhang and Tian 2009). Functional cell membranes prevented cell collapse and therefore hot water dips were effective in mold control due to elimination of a favorable environment for their growth.

## Advanced Processing Technologies

In recent years, a number of novel, alternative, or “advanced” processing technologies have generated a lot of interest for their ability to insure microbiologically safe products with long shelf life and superior quality as compared to conventional thermally processed foods. Many of these technologies were initially classified as “nonthermal,” although heat may still be generated during application of the processes. In general, the temperatures to which foods are exposed in these advanced processes are relatively low and may be below pasteurization temperatures (Butz and others 2002; Gerlach and others 2008; Oey and others 2008). For this reason, there is tremendous potential for production of superior quality food products. In general, heat adversely affects texture, color, flavor, and nutrient content. Foods can be processed by methods such as irradiation, high hydrostatic pressure, ultrasound, filtration, use of antimicrobials, and electrical methods such as pulsed electric fields (PEFs), ohmic, microwave, radiofrequency, light pulses, and oscillating magnetic fields. These methods are attractive to the food industry because more fresh-like, flavorful, colorful, and nutrient rich may be produced.

### 

#### HP processing

**Figure 3:**
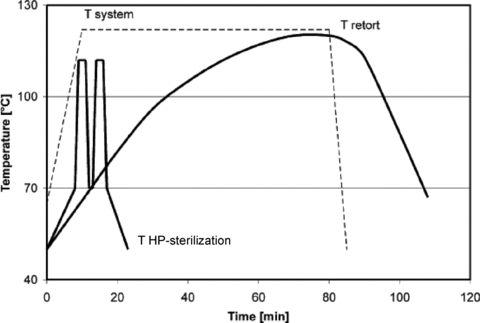
Temperature in center of can with spinach (T retort) compared to temperature of the retort unit (T system). Temperature in center of pouch with spinach during high-pressure sterilization (T HP-sterilization). (From Matser and others 2004.)

High hydrostatic pressure processing is the advanced technology that is being adopted most quickly by the food industry as a potential alternative to pasteurization of food products (Basak and Ramaswamy 1998; Welti-Chanes and others 2005; Rastogi and others 2007). Recently combination HP high temperature processes are also being studied as sterilization processes (Matser and others 2004; Rastogi and others 2008). HPs range from 100 MPa (c. 1000 atm) up to 900 MPa (c. 9000 atm), and pressures used in commercial systems commonly are between 400 and 700 MPa (San Martin and others 2002). The extent of temperature increase during pressure application varies with the composition of the food but is normally 3 to 9 °C/100 MPa (Patterson 2005). Examples of successful HP-treated foods commercially available are fruit jams and sauces (Cano and de Ancos 2005), guacamole, sliced cooked hams, oysters, and meal kits that contain meat, salsa, guacamole peppers, and onions (Patterson 2005). Matser and others (2004) illustrated that temperature exposure during HP processing was much lower than conventional heat sterilization (Figure 3).

### Effects of HP on biochemical reactions

Most biochemical reactions result in a volume change and are therefore affected by pressure (Patterson 2005). HP treatments favor biochemical reactions that lead to a volume decrease while inhibiting or retarding reactions that lead to a volume increase. Noncovalent interactions constitute the main target for the modulation of biomolecular characteristics through pressure (Balny 2004). Application of HP to food products results in increased microbial inactivation, promotes protein conformational changes, and may enhance enzyme inactivation (Barbosa-Cánovas and others 2005). Enzymes vary greatly in their ability to withstand pressure (Patterson 2005), with some enzymes activated and others inactivated by HP.

Boonyaratanakornkit and others (2002) reported that the effects of pressure on protein structure and function can vary dramatically depending on the magnitude of the pressure, the reaction mechanism (in the case of enzymes), and the overall balance of forces responsible for maintaining protein structure. Also, interactions between the protein and the solvent are critical in determining the response of a protein to pressure. Most covalent bonds participating in the protein primary structure are pressure insensitive, at least up to 1000 to 1500 MPa. Thus, in the absence of covalent bond formation or breaking, the largest contributions are expected to arise from hydration changes that accompany noncovalent interactions (Balny 2004). Covalent bonds are negligibly compressible under pressure and are generally unaffected at the pressures used in food processing. This means that many of the components responsible for the sensory and nutritional quality of foods, such as flavor components and vitamins, are not destroyed by HP (Patterson 2005), making this technology of interest to the food industry.

Kato and others (2002) showed in a specific study involving membrane bound Na^+^/K^+^ ATPase that HP effects on membrane damage take place in 3 steps. Pressures below 100 MPa induced a decrease in membrane fluidity of the lipid bilayer and reversible changes in transmembrane protein conformation. Pressures of 100 to 220 MPa caused reversible phase transitions in parts of the lipid bilayer, from the liquid crystalline to the gel phase, and dissociation and conformational changes in the protein subunits. Pressures above 220 MPa destroyed and fragmented the membrane structure due to protein unfolding and interface separation. These changes explain pressure observed damage to cell organelles at approximately 200 to 300 MPa in plant cells and microorganisms. Tauc and others (1998) observed that HP increases the orientational ordering of fatty acids, no matter whether they are saturated or unsaturated, while temperature decreases the ordering.

### Effects of HP on microorganisms

Yeast cells are interesting to compare to plant cells because of the presence of a cell wall, a central vacuole, and organelles. Most microbial cells lack a cell wall, but yeast cells are more similar to plant cells. Even though the vacuolar composition in yeasts is mainly lipidic (Hartmann and others 2006), the presence of similar structures makes it an interesting case for study with respect to plant cells. In yeast cells, no alterations in the subcellular structure were observed below 100 MPa, but at around 200 MPa transmission electron microscopy results indicated an alteration in subcellular structure, where the nucleus membrane pores were shown and morphological changes in mitochondria were observed, resulting in the yeast cells being incapable of growth (Sato and others 1995).

Hartmann and Delgado (2004) studied the mechanical effects of compression in yeast cells by modeling and simulation, taking into account material parameters derived from thermodynamic relationships of water and lipids under high hydrostatic pressure. It was found that the deformation of the cell under pressure deviates strongly from isotropic volume reduction, and in particular organelle membranes exhibit 80% effective strain value at 400 MPa. These authors showed that the presence of different material resistances in a cell generated a heterogeneous distribution of strains with consequently substantial deformations, with excessive strain on organelle membranes and excessive stress in the cell wall, and concluded that high hydrostatic pressure treatment may mechanically injure biological cells and tissues.

In bacteria, *Lactobacillus plantarum* showed inactivation of the membrane transport system at relatively low pressures of 200 MPa, which represented a sublethal injury but did not affect the viability of the cell (Gänzle and others 2001). In *Escherichia coli*, Manas and Mackey (2004) determined that the pressure resistance of the stationary phase was much higher than that of exponential phase cells, both types presenting aggregation of cytoplasmic proteins and condensation of the nucleoid after treatment at 200 MPa for 8 min. In addition to these events, exponential phase cells showed perturbations of envelope structure, loss of osmotic responsiveness, and loss of protein and RNA to the extracellular medium. Based on this evidence, the authors proposed that exponential phase cells were inactivated under HP by irreversible damage to the cell membrane. In *E. coli*, Casadei and others (2002) determined that there was a relationship between culture growth temperature, membrane fatty acid composition, and pressure resistance in exponential and stationary phase cells. Deep-sea bacteria have been studied to understand the adaptive changes in response to the increase in pressure. It was found that a greater amount of unsaturated fatty acids in the membrane lipids was present (DeLong and Yayanos 1985; Yano and others 1998). In *S. cerevisiae*, mutant strains that lacked the ability to accumulate trehalose, a molecule that can play a role in stabilizing membranes (Crowe and others 2001), and/or accumulate heat shock proteins showed less barotolerance than the control strain when exposed to 180 MPa (Iwahashi and others 1997).

### Effects of HP on plant tissues

Changes in cell biopolymers (proteins, polysaccharides, and lipids) occur during HP treatments. Pressure induces changes in polysaccharides, which can affect their functionality and the texture/structure of plant foods (Butz and others 2002; Butz and Tauscher 2002; Cano and de Ancos 2005). Protein structure (unfolding, aggregation, gelation) and fat crystallization have been shown to take place as a result of HP treatment.

Textural changes in plant tissues caused by pressure treatments may result from physical disruption of the tissue. Prestamo and Arroyo (1998) observed cellular structure changes and membrane folding of cauliflower and spinach leaves after HP processing at 400 MPa with cryofracture scanning electron microscopy. Microscopic studies of onion epidermis cells revealed severe damage to the vacuoles after 300 MPa treatments at 25 °C, with the odor of fresh onions changed toward that of braised or fried onions, and a strong increase in 2-methyl-pent-2-enal, one of the main products of alliinase (Butz and others 1994). Luscher and others (2005) stated that the membrane damage extent of vegetable tissue might be influenced by the relative rigidity of the gel-phase membranes and that a better understanding of the state of the membranes after the pressure treatments, with or without phase transitions, might give an explanation for the extent of cell viability, drip loss, and changes in texture related to turgor pressure in plant tissue materials. Measurement of the maximum photochemical efficiency in lettuce, which is a physiological indicator of photosynthetic activity and therefore cell viability and tissue vitality (Schlüter and others 2008), showed that HP treatments of 150 MPa caused irreversible cell damage, and critical changes in the chloroplastic membrane integrity. Below 150 MPa, transient changes in the membrane can be inferred since there was an initial decrease in maximum photochemical efficiency, with recovery observed during a 24 h period. Cell damage in this study was affected by pressure level and treatment durations.

Kato and others (1997) studied the effect of HP on pectic substance degradation and tissue softening. No degradation of pectic substance was observed after 45 min at 700 MPa. However, the degree of esterification decreased in pressurized carrot disks, suggesting pectin methyl esterase activity occurred. Similar results were observed by De Roeck and others (2008), following the molar mass distribution of pectin polysaccharides in carrots in brine treated at high temperature and atmospheric pressure (80 °C, 0.1 MPa) and under a HP sterilization treatment (80 °C, 600 MPa). Their results indicated that solubilization of cell wall components occurred as a result of high-temperature processing, whereas the combination of high temperature and pressure processing did not solubilize the cell wall.

Butz and others (2002) determined that vegetable matrices had altered water retention after HP treatments, where the water release of tomato pulp measured after centrifugation indicated that 600 MPa, 60 min treated samples had much lower water release than the 95 °C, 60 min, and the untreated samples. This change in the water release observed was attributed to the strong effect HP has on the structure of macromolecules, affecting binding properties of polar and nonpolar substances.

Roldán-Marín and others (2009) showed that the processing of onion (*Allium cepa* L. var. *cepa*, “Grano de Oro”) with treatments that combine low temperature (5 °C) with pressures of 100 and 400 MPa for a constant time (5 min) significantly increased the amount of total phenols extracted from onion. Low temperature pressure processing resulted in an increase in quercetin-4′-glucoside, total quercetin, and quercetin-3,4′-diglucoside yields of 33, 26, and 17%, respectively, as compared with untreated onions. Moreover, processing onions at low (5 °C) and medium (27.5 °C) temperatures, combined with a HP of 400 MPa maintained the antioxidant activity of the untreated onions and there was a trend toward an increase in antioxidant activity in pressurized onions as pressure levels were raised from 100 to 400 MPa. Disruption of cellular compartmentalization may be desired, as it may lead to improved bioaccesability (Verlinde and others 2008) and extraction yield (Oey and others 2008) of certain nutrients.

#### Electric field processing

**Figure 4:**
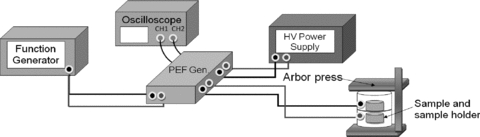
Schematic diagram of the pulsed electric field treatment system.

Over the past 20 y, there has been a tremendous increase in the published literature related to the potential use of different parts of the electromagnetic spectrum, such as ohmic, moderate and PEFs, infrared and microwaves, to process foods. In general, field strengths of *E* < 100 V/cm are considered to be low intensity electric fields, while *E* in the range of 0.1 to 1 kV/cm are considered to be moderate electric fields, and *E* > 5 kV/cm are considered to be high-intensity electric fields (Fincan and Dejmek 2002; Lebovka and others 2002; Rastogi 2003; Loeffler 2006). A typical setup for PEF applications includes a power supply, function and pulse generator, sample chamber with 2 electrodes, and data acquisition system such as depicted in Figure 4 (Asavasanti and others 2010).

As with HP applications, these methods may hold promise for production of higher quality preserved foods due to their inherent ability to uniformly and simultaneously treat the entire food.

### Electric field effects on microorganisms

In a review by Raso and Barbosa-Canovas (2003), the authors stated that PEFs are very effective at killing vegetative cells of bacteria, yeast, and mold but may be less effective at destroying spores and enzymes. Application of high-intensity electric fields results in increased permeability of microbial and plant cell membranes, creating reversible, and/or irreversible pores in the primarily lipid membrane structure. For microbial cells, this quickly leads to cell death. The mechanism by which PEFs inactivate microorganisms is not completely understood, but Heinz and others (2002) suggested that damage to the cell membrane is the primary response causing microorganisms to die following PEF processing. These authors described a critical level of PEF application above which microbial inactivation occurred.

Previous studies have found that plant tissue disruption under PEF treatments can be achieved at room temperature using moderate electric fields of 0.5 to 5.0 kV/cm within 10^−4^ to 10^−2^ s, whereas for breakdown of microbial membranes, field strengths of 15 kV/cm, and higher are required (Dunn 2001; Lebovka and others 2001, 2002). Wouters and others (2001) found a linear relationship between microbial inactivation and percentage of permeabilized cells, up to a 3.6 log reduction, using electric fields between 12 and 15 kV/cm. These authors stated that the primary process parameters that affect microbial inactivation are the electric field strength, number of pulses, duration and shape of pulses, and initial product temperature.

### Electric field effects on plant tissues

Knorr and others (2001) reviewed food processing scenarios that would benefit from the application of PEFs to increase mass transfer and therefore assist with osmotic dehydration or expression of plant cell extracts. These authors described the effects of high electric field pulses (HELP) on various plant tissues, as illustrated in Table 1. Because electric fields increase the permeability of membranes that retain the primarily liquid cell contents, they create larger openings through which water can be diffused to dehydrate and concentrate plant tissues, and also if these openings are reversible they provide for easier introduction of desirable components such as nutrients or flavor compounds.

**Table 1:** Changes in fruit juice composition following pre-treatments with pulsed electric fields^1^ (adapted from Knorr and others 2001).

**Compositional factor**	**Grapes**	**Apple**	**Black currant**
Total solids (°Brix)	Higher	Higher	Same
Density (g/mL)	Higher	Same	Same
Acidity (meq/L)	Higher	Higher	Higher
pH	Lower	Higher	Same
Conductivity (ms/cm)	Higher	Higher	Higher
Turbidity	Lower	Higher	Lower
Pectins	Lower	Higher	Higher
Proteins	Higher	Same	Higher
Ascorbic acid		Higher	

1 PEF treatments were 2 to 3 kV/cm, 20 to 40 pulses at the rate of 0.7 ms.

Most commercial PEF applications to date have been on liquid products such as juices, but there is 1 commercial operation that utilizes PEF for extraction of beet sugar (Heinz and others 2002). Angersbach and others (1999) have suggested that PEF treatment of plant tissues can initiate separate membrane breakdowns of the plasma membrane and the tonoplast membrane, giving rise to 2 possible critical electrical field strengths. However, they did not present data to support their hypothesis. Application of PEFs of sufficient strength and pulse number results in not only membrane changes but also removal of the turgor component of texture (Lebovka and others 2003). Lebovka and others (2004) studied the effect of PEF treatments on textural properties of carrots, potatoes, and apples, reporting that PEF resulted in loss of turgor and rupture of cell membranes.

## Quantification of Cell Membrane Permeability and/or Integrity in Plant Tissues

In plants, cell membranes are one of the first targets of plant stress, and alterations in membrane structure may cause a modification of cellular compartmentalization (Vazquez-Tello and others 1990). From a biological point of view, mild processing of plant tissue will mimic stress conditions, therefore knowledge of how the plant material will be affected in relation to different food processing manipulations is fundamental for quality assurance and process optimization (Gomez Galindo and others 2007). The quantification of cellular disruption in plant tissues, imparted by the loss of membrane integrity, will allow correlate the cell structure changes occurring at the molecular and microscopic level to the functionality and quality of fruit and vegetable products (Knorr 1994; Angersbach and others 1999). Cell membrane deterioration may be assessed in many different ways, for example, changes in composition, structure, or function such as fluidity or permeability, or by the loss of protein functionality. In living cells and multicellular tissues, membrane permeability has previously been estimated using a number of methods, including measuring conductivity of leachates and solids lost during soaking and volume exudates (Vasquez-Tello and others 1990), light scattering, fluorescence microscopy and volume-sensitive fluorescent indicators (Stanley 1991; Verkman 2000), electrical impedance (Rastogi and others 1999), and nuclear magnetic resonance (Van Der Weerd and others 2001, 2002). Some of the methods that have been used in plant tissues to test for cell membrane permeability and/or integrity in intact and processed plant tissues are briefly described below.

### Electrolyte leakage

The amount of ion efflux into a solution plant tissue is immersed in has long been used as a measurement of the intactness and permeability of cell membranes (Murray and others 1989; Vasquez-Tello and others 1990). The relationship between conductivity (the electrolyte concentration in solution) and time has been shown to follow an asymptotic curve and may be represented by a 1st-order reaction equation, where the rate of leakage varies with the extent of tissue damage (Murray and others 1989). The initial fast increase in conductivity has been associated with passive physicochemical processes (diffusion, adsorption/desorption) in the apoplast, while the 2nd slower stage is attributed to the functional activity of the plasmalemma (Kocheva and others 2005; Saltveit 2002). Increased injury, as indicated by the net leakage, may result from either an increased efflux due to damage to the semipermeability of the plasmalemma, or a decreased influx due to damage to the active transport system (Palta and others 1977). Lack of selectivity regarding the contribution of different ions, as well as the interpretation in terms of physicochemical and electrochemical parameters of the membranes are some of the limitations to this method (Kocheva and others 2005).

### Cell viability

The determination of viable cells by membrane integrity assays rely on the uptake and active retention of dyes such as neutral red (Admon and Jacoby 1980; Ehara and others 1996) and fluorescein (Heslop-Harrison and Heslop-Harrison 1970) in living cells, or passive staining of the contents of dead cells with dyes such as Evans blue, that leaks through ruptured cells (Baker and others 1994). The FDA method as described by Heslop-Harrison and Heslop-Harrison (1970) has been used for fleshy fruit tissues in developing grapes (Krasnow and others 2008) and in cucumber tissue (Sajnín and others 2003). This method detects active cellular metabolism by the conversion of FDA, a nonpolar nonfluorescent fluorescein analogue that passes through the cell membrane, whereupon intracellular esterases cleave off the diacetate group, producing the highly fluorescent product, which accumulates in cells with intact membranes. Fluorescent dyes have also been used for microorganisms, where, for example, flow cytometric analysis has been applied to study cell membrane site injuries to identify viability of bacterial populations (Ananta and others 2005).

A nonfluorescent dye, widely used for cell viability and plant cell vacuole staining (Admon and Jacoby 1980) is neutral red. This azine dye is uncharged and nonionized in alkaline solutions, diffuses across membranes due to its lipophilic nature and ionizes and accumulates in the acidic vacuolar medium, appearing as dark red colored vacuoles in intact cells (Ehara and others 1996; Fincan and Dejmek 2002). The penetration of the dye into the tissue depends on the integrity of the cell membrane and the capacity to maintain pH gradients (Repetto and others 2008).

### ^1^H-NMR

The application of NMR imaging and relaxometry studies of plants subjected to stress has proven to be a valuable technique for reflecting anatomical details of the entire tissue and water status (Van Der Weerd and others 2001, 2002). ^1^H-NMR studies on intact plant tissues have shown that spin lattice (T_1_) and spin–spin (T_2_) relaxation times can be related to the water content of the tissue, the properties of water in different parts of the tissue and the interaction with macromolecules, discriminating different populations of water within the tissue. The exchange rates between the cellular compartments are controlled by the permeability of the intervening membranes (Snaar and Van As 1992a, 1992b; Van Der Weerd and others 2002). Protons with a short relaxation time are associated with the extracellular (apoplastic) water and with the total tissue water of hydration, while protons with a long relaxation time are associated with the intracellular water (Snaar and Van As, 1992b; Van Der Weerd and others 2002). A loss of cellular compartmentalization results in an exchange of water protons between the less mobile extracellular water as well as the tissue water of hydration and the more mobile intracellular water, contributing to the decline in the spin–spin relaxation time, T_2_ (Hills and Remigereau 1997; Maheswari and others 1999).

^1^H-NMR diffusion experiments can also be used to determine properties of the cell boundaries (Anisimov and others 1998; Ionenko and others 2006). The bulk diffusion coefficient depends on the temperature and viscosity of the fluid as well as the boundaries encountered that will restrict diffusion of the water molecules (Van As 2007). Ionenko and Anisimov (2001) demonstrated with a spin-echo NMR method that the roots of maize seedlings exposed to treatments that destroyed cell membranes, for example, nitrogen vapurs, boiling water vapur, diethyl ether and low temperature (−10 °C), had an increase in the diffusion coefficient with respect to the untreated roots. Using T_2_-diffusion correlation spectroscopy in pears, Hernández-Sánchez and others (2007) found that fruit with internal browning, a disorder that developed during storage in controlled atmosphere that leads to cell de-compartmentalization and browning reactions, had higher diffusion coefficients of the 2 compartments determined than those of the sound tissue.

## Research Approach

The accompanying 6 manuscripts aim to understand the effects on plant tissues of HP and electric field processing, 2 of the most prominent new technologies being quickly adopted by the food industry. Cell membranes are one of the 1st targets of plant stress and many food processes impact membrane integrity, causing detrimental quality as a result of undesired biochemical reactions and loss of texture attributes. Different methods, of different complexity and accessibility, that allow quantification of changes in membrane permeability and integrity were evaluated and related to texture changes in onions after HP, electric field, and thermal processing.

The manuscripts by Gonzalez and others (2010a) as well as by Gonzalez and others (2010b) are an anatomical and cytological approach to cell integrity quantification. Neutral red, a dye commonly used to evaluate cell viability and integrity of plant vacuoles was used. Image analysis was used as a quantification methodology. In the 2nd manuscript, texture analysis of raw and processed samples was carried out and the different texture parameters studied were correlated to cell membrane integrity.

In the manuscript by Gonzalez and others (2010c)^1^H-NMR was used as a quantification method to study cell integrity after HP and thermal processing. ^1^H-NMR relaxometry was used as a tool to determine the changes in the different proton environments within the raw and processed tissue and obtain information on cell compartmentalization.

The 4th manuscript presents a biochemical approach by Gonzalez and others (2010d) and the products of enzymatic reactions, which are formed after loss of cell of compartmentalization, were monitored as indicators of cell rupture. The leakage of electrolytes into solution, frequently used to evaluate membrane damage, was compared. An integration of all methods was used to determine changes in membrane permeability and integrity. There was strong agreement between methods in the determination of the ranges of HP and temperature that induce changes at the plasmalemma and tonoplast level.

The 5th and 6th manuscripts in this series evaluate another method of minimal processing, PEFs, using the same model onion tissue, and many of the same methods of quantifying effects on membrane integrity. The manuscript by Asavasanti and others (2010) measures the electrical properties, ion leakage rate, texture and amount of enzymatically formed pyruvate before and after PEF treatment for a range of applied field strengths and number of pulses. The last manuscript, by Ersus and others (2010), uses ^1^H-NMR and ion leakage to evaluate the effects of electric field strength, pulse width, total pulse duration, and frequency on the integrity of onion tissues.

## Conclusions

The quantification of changes that plant tissues undergo at the macroscopic, microscopic, and molecular level as a result of food processing will allow for a better comprehension of how tissue structure impacts the texture, color, flavor, and nutrient content of fruit and vegetable products. This review emphasizes the effects of HP and electric field processing, 2 of the most prominent new technologies being quickly adopted by the food industry, and their impact on biological tissues as compared to thermal processes. Plant cell membranes are one of the 1st targets of plant stress, where small increases in the level of physical stress applied, may make the difference between reversible and irreversible membrane changes. Many food processes are above these threshold limits and impact membrane integrity, causing detrimental loss of texture attributes and affecting their “fresh-like” quality. Some methods that will allow for cell membrane integrity quantification in plant tissues were discussed and were evaluated in the accompanying manuscripts, where the effects of HP, pulse electric filed, and thermal processing on cell membrane integrity at the tonoplast and plasmalemma level were determined.
